# Novel protein acetyltransferase, Rv2170, modulates carbon and energy metabolism in *Mycobacterium tuberculosis*

**DOI:** 10.1038/s41598-017-00067-1

**Published:** 2017-03-06

**Authors:** Wonsik Lee, Brian C. VanderVen, Suzanne Walker, David G. Russell

**Affiliations:** 1 Department of Microbiology and Immunology, College of Veterinary Medicine, Cornell University, Ithaca, NY 14853 United States; 2 Department of Microbiology and Immunology, Harvard Medical School, Boston, Massachusetts 02115 United States

## Abstract

Recent data indicate that the metabolism of *Mycobacterium tuberculosis* (Mtb) inside its host cell is heavily dependent on cholesterol and fatty acids. Mtb exhibits a unique capacity to co-metabolize different carbon sources and the products from these substrates are compartmentalized metabolically. Isocitrate lies at one of the key nodes of carbon metabolism and can feed into either the glyoxylate shunt (via isocitrate lyase) or the TCA cycle (via isocitrate dehydrogenase (ICDH) activity) and we sought to better understand the regulation at this junction. An isocitrate lyase-deficient mutant of Mtb (*Δicl1*) exhibited a delayed growth phenotype in stearic acid (C18 fatty acid) media and we isolated rescue mutants that had lost this growth delay. We found that mutations in the gene *rv2170* promoted Mtb replication under these conditions and rescued the growth delay in a *Δicl1* background. The Mtb Rv2170 protein shows lysine acetyltransferase activity, which is capable of post-translationally modifying lysine residues of the ICDH protein leading to a reduction in its enzymatic activity. Our data show that contrary to most bacteria that regulate ICDH activity through phosphorylation, Mtb is capable of regulating ICDH activity by acetylation. This mechanism of regulation is similar to that utilized for mammalian mitochondrial ICDH.

## Introduction

*Mycobacterium tuberculosis* (Mtb) is a metabolically-flexible bacillus that is capable of co-metabolizing simple carbon substrates. It is this metabolic plasticity that is believed to be a major factor in the success of this pathogen within its target host species, mankind^1, 2^. During infection, Mtb experiences a range of environments within the granulomas formed at site of infection^3–5^. The environmental heterogeneity within the granuloma likely presents certain challenges to Mtb through the variation in oxygen tension and the availability of nutrients, which appear limited in diversity if not always in abundance. To adapt to these environments, Mtb efficiently reprograms its metabolism^6^, most notably towards the effective utilization of host-derived fatty acids and cholesterol^7, 8^ as the primary carbon source(s) exploited *in vivo*.

Most bacteria regulate their metabolism through carbon catabolite repression (CCR)^9^, which results in diauxic growth as preferred carbon sources are individually and sequentially depleted from the medium. From an evolutionary standpoint this form of regulation would confer an advantage on bacteria in a competitive environment where speed of growth on a preferred carbon substrate would afford a distinct benefit. In contrast, Mtb within its host does not have to out-compete other bacteria and has evolved a different strategy for nutrient utilization that enables it to metabolize a range of different carbon sources. Intriguingly, despite the fact that carbon substrates have the capacity to generate the same metabolic intermediates, analysis with simple carbon substrates such as acetate, dextrose, and glycerol indicate that the utilization of carbon from these substrates is highly compartmentalized and feeds into different downstream metabolic pathways^2^.

In the absence of the top-down regulatory controls, Mtb has evolved other mechanisms to modulate carbon flux at critical metabolic nodes. Mtb exhibits two distinct routes of carbon flow according to a given carbon source; In one, glycolytic carbon substrates such as glucose can fuel the TCA cycle, generating a full set of intermediates plus energy and releasing 2 moles of carbon dioxide. In the other, gluconeogenic carbon substrate such as acetate or fatty acids, flow into the glyoxylate shunt pathway to replenish the four carbon intermediates succinate and malate, thus avoiding carbon loss as carbon dioxide through the activities of isocitrate dehydrogenase (ICDH) and α-ketoglutarate dehydrogenase in the TCA cycle. Isocitrate, the substrate of isocitrate lyase (ICL), lies at a branch point between the TCA cycle and glyoxylate shunt. In this branching metabolic node, ICDH competes with ICL for their substrate, isocitrate. Because the substrate affinity of ICDH for isocitrate is higher than that of ICL, flux at this metabolic node is highly dependent on factors that affect the activity of ICDH. For example, in *Escherichia coli*, the *Km* (604 μM) of ICL is much higher than that of ICDH (8 μM)^10, 11^. Similarly, in Mtb the *Km* (145 μM) of ICL1 (Rv0467) is higher than that of ICDH1 (Rv3339c) (10 μM)^12, 13^, implying that Mtb may be regulated at the isocitrate node in a manner analogous to *E. coli*. In *E. coli*, ICDH activity and the flow of carbon from isocitrate into the TCA cycle is modulated by post-translational modification through phosphorylation of ICDH by AceK^14^. Mtb, however, lacks any homologues to *E. coli* AceK, indicating that Mtb likely employs a different strategy for ICDH regulation at the isocitrate node^15^.

In this current study we probed the regulation of ICL1 and ICDH1 activities and took an advantage of a growth defect phenotype in an Mtb *Δicl1* mutant when grown on fatty acids as a sole carbon source. We exploited this phenotype to conduct a forward genetic screen to identify mutants that restored growth in an Mtb *Δicl1* mutant, presumably through increasing carbon flow through ICDH and α-ketoglutarate dehydrogenase in the TCA cycle. One gene identified encoded a novel GCN5-like protein acetyltransferase (GNAT) (Rv2170), which we hypothesized is involved in post-translational regulation of ICDH1. The transposon insertion in this gene restored the ability of an Mtb *Δicl1* mutant to grow on stearic acid, and an episomally-encoded copy of Rv2170 reverted the mutant back to the parental null-growth phenotype. Furthermore, recombinant, Rv2170 protein acetylated recombinant ICDH1, which down-regulated its enzymatic activity. *In vitro* acetyltransferase assays and LC-MS/MS analysis confirmed acetylation of recombinant ICDH1 and identified two residues, Lys30 and Lys129 as the sites of acetylation. These data identify Rv2170 as a novel regulator of carbon flux into the TCA cycle and provide mechanistic insights into the regulation of central carbon metabolism in Mtb. In addition, this post-translational modification by acetylation on ICDH1 may be broadly applicable to other bacterial NADP^+^-dependent ICDHs that are structurally homologous to Mtb’s ICDH1.

## Experimental Procedures

### Bacterial strains and growth conditions

Bacterial strains, plasmids, and primers are listed in the supplemental material (Supplementary Tables 2 and 3). Wild type *M. tuberculosis* (Mtb) H37Rv and derivatives were maintained aerobically at 37 °C in Middlebrook 7H9 medium or 7H10 supplemented with 0.2% glycerol, 10% OADC (0.5 g/L oleic acid, 50 g/L albumin, 20 g/L dextrose, and 0.04 g/L catalase) and 0.05% tyloxapol. For sole carbon source experiments, Mtb strains were grown in modified Sauton’s medium containing 0.5 g/L (NH_4_)_2_SO_4_, 0.15 g/L asparagine, 1.0 g/L KH_2_PO_4_, 2.5 g/L Na_2_HPO_4_, 50 mg/L ferric ammonium citrate, 0.5 g/L MgSO_4_ 7H2O, 0.5 mg/L CaCl_2_ and 0.1 mg/L ZnSO_4_, and 0.05% tyloxapol supplemented with 10 mM glycerol or 0.05 mM stearic acid as described^7^. *Escherichia coli* TOP10 or BL21 (DE3) were used for gene cloning and maintained at 37 °C in LB broth or LB agar plates. For Mtb strains, kanamycin (20 μg/mL) or hygromycin (50 μg/mL), were added as needed. Ampicillin (100 μg/mL) was used for *E. coli* selection. Bacterial growth was monitored by measuring optical density at 600 nm.

### Screen transposon mutants and identification of transposon insertions

To select transposon mutants that are able to grow on stearic acid, we generated a library of 1 × 10^5^ transposon mutants in the *Δicl1* H37Rv background as described^7^. The transposon library was washed 3 times with phosphate-buffered saline (PBS) and inoculated on the minimal medium containing 0.05 mM stearic acid as sole carbon source to an initial OD_600_ of 0.01. When the culture reached OD_600_ 0.3, we plated the culture onto 7H10 OADC agar supplemented with 20 μg/mL kanamycin and 50 μg/mL hygromycin and incubated on 37 °C. Colonies were selected and retested to confirm their growth phenotype on the plate. Genomic DNA was extracted from the individual mutants and the transposon insertion sites were amplified by PCR and sequenced as described^16^.

### Generation of Rv2170 knockout and complementation strains

To construct *ΔRv2170* mutant in H37Rv, we performed gene replacement with hygromycin resistant cassette as described^17^. A flanking region of Rv2170 (approximately 1000 bp) was PCR amplified from genomic DNA of wild type H37Rv. Then, the PCR products were cloned into the pYUB854-*rspL* in the flanking region of the hygromycin resistance cassette. This plasmid was UV irradiated and electroporated into a streptomycin resistant H37Rv rpsL mutant. Transformants were selected on 7H10 agar OADC plate containing 100 μg/mL streptomycin and 100 μg/mL hygromycin. Putative transformants resistant to both streptomycin and hygromycin were screened by PCR for correct allelic exchange using primers external to the homologous flanking regions and internal to hygromycin cassette, and further confirmed by sequencing. To construct the complementation strain, the Rv2170 gene was PCR amplified and cloned into the plasmid pVV16 under the hsp60 promoter, which expresses the gene constitutively. The complementation plasmid was then transformed into the *ΔRv2170* mutant.

### Rv2170 protein sequence alignment and phylogenetic analysis

The amino acid sequence of Mtb Rv2170 (NP_216686.1) was blasted against the NCBI protein database. For phylogenetic analysis, 13 representative sequences were selected: *M. smegmatis* (YP_888515.1), *M. avium* (ABK64727.1), *M. marinum* (ACC41631.1), *M. abscessus* (YP_001702731.1), *M. vanbaalenii* (ABM14329.1), *M. ulcerans* (ABL05668.1), *M. leprae* (WP_010908010.1) *Corynebacterium glutamicum* (NP_601374.1), *C. diphtheriae* (WP_010935193.1) *C. efficiens* (BAC18875.1) *Streptomyces coelicolor* (NP_629826.1) *Nordia farcinica* (WP_011208285.1), *Rhodococcus jostii* (ABG92927.1). The 14 multiple sequences were aligned with MUSCLE program^18^. The aligned sequences then were used to generate phylogenetic tree using PhyML and TreeDyn. Boot analysis was used on statistical test for branch support.

### ^14^C-radiorespirometry assay

To determine metabolic activity of Mtb strains, we measured oxidation of stearic acid by monitoring the released ^14^CO_2_ from [1-^14^C] stearic acid (PerkinElmer Life Sciences), which was the sole carbon source as described^19^. Briefly, Mtb strains grown in minimal medium containing 10 mM glycerol were washed 3 times with PBS and inoculated into 10 mL of minimal medium containing 0.05 mM stearic acid and 1 μCi/mL [1-^14^C] stearic acid to an initial OD_600_ of 0.1 in vented T-25 tissue culture flask. Each flask was placed in an air-tight jar with an open vial of 500 μL of 1N NaOH, and incubated at 37 °C. For each time point, the vial of NaOH was removed and neutralized with an equal volume of 1 N HCl. The amount of solubilized ^14^CO_3_ was quantified by scintillation counting (Beckman Coulter LS6500). Total CPM (counting per minutes) was normalized by dividing by the OD_600_ at each time point.

### Overexpression and purification of Rv2170 and ICDH1 proteins

Rv2170 and ICDH1 were PCR amplified from H37Rv genomic DNA and cloned into an over-expression plasmid pET23a (Novagen) for synthesis of proteins with a C-terminal 6-His tag. The DNA sequences of Rv2170 and ICDH1 were confirmed by sequencing. The constructed plasmids were transformed into a T7-express *E. coli* strain BL21 DE3 (Novagen). A 5 mL overnight culture from a single colony was inoculated in 500 mL of LB containing 100 μg/mL ampicillin. The cells were grown to early log phase (OD_600_ = ~0.3) at 37 °C while shaking at 200 rpm and then induced with 1 mM isopropyl β-D-1-thiogalactopyranoside (IPTG) at 16 °C for 20 hours with shaking at 100 rpm. Bacterial cells were harvested by centrifugation at 4000 rpm for 15 min and resuspended in 5 mL ice cold lysis buffer (300 mM NaCl, 50 mM Tris-HCl pH7.5) containing protease inhibitor cocktail (Sigma, P8849), 0.5mg/mL lysozme and DNase I. Cells were incubated on ice for 30 minutes and lysed on ice using 505 Sonic Dismembrator (Fisher Scientific) for 2 minutes, followed by centrifugation at 30,000 × g for 30 minutes at 4 °C. Clarified cell extracts were loaded onto a 1-mL bed volume of Talon Metal affinity resin (Clontech). After incubation for 30 minutes at room temperature, the column was washed with a wash buffer (300 mM NaCl, 50 mM Tris-HCl pH 7.5, 20 mM imidazole). The proteins were eluted off the column with an elution buffer (300 mM NaCl, 50 mM Tris-HCl pH 7.5, 250 mM imidazole). The eluted fractions were analyzed by SDS PAGE, and the fractions containing protein of the correct mass were pooled and dialyzed against a buffer containing 150 mM NaCl, 50 mM Tris-HCl pH 7.5. The ICDH1 was verified identify by western blot using an anti-his tag antibody.

### Rv2170 *in vitro* activity assay

#### Acetyltransferase assay with ^14^C radioactive CoA-substrate

ICDH1 acetylation by Rv2170 detected using radiolabeled acetyl-CoA as described^20^. Assays were carried out in 100 μL total reaction mixture containing 50 mM pH 7.0 HEPES, 50 μM ^14^C radioactive CoA-substrate ([1, 2-^14^C]acetyl-CoA, [1-^14^C]propionyl-CoA, and [1-^14^C]succinyl-CoA from American Radiolabeled Chemicals), 20 μg Rv2170 (acetyltransferase), and 20 μg ICDH1. Reactions were incubated for 2 hours at 30 °C and stopped with a 5× gel-loading buffer (250 mM Tris-HCl, pH6.8, 30% glycerol, 10% SDS, 0.02% bromophenol blue, 5% β-mercaptoethanol). Samples were resolved using SDS-PAGE and visualized by Coomassie Blue staining. The SDS-PAGE gel was exposed to a phosphor screen for 24 hours. The radioactivity was detected using a phosphoimager (Storm Imager).

#### Acetyltransferase assay with DTNB

The acetyltransferase activity was determined by free CoA by using DTNB (5,5-dithio-bis-(2-nitrobenzoic acid) which reacts with sulfhydryl group to give a yellow product, 2-nitro-5-thiobenzoate as describe^21^. Assays were performed at 37 °C in 100 μL volume on a black clear bottom 96 well plate (Corning). Reaction mixtures contained 50 mM HEPES buffer (pH 7.5), 0.3 mM DTNB, acetyl-CoA, Rv2170 and ICDH1. Assays were initiated by adding Rv2170 (10 μg) enzyme. Substrate saturation curves were performed either with acetyl-CoA fixed at a saturating concentration (200 μM) while ICDH1 concentrations were varied from 0 to 17 μg or with the ICDH1 concentration fixed at 10 μg and the acetyl-CoA concentration varied from 3.5 to 50 μM. Data were collected every 10 s over a 30-min time period at 412 nm using a Perkin-Elmer EnVision microtiter plate reader. All measurements were performed in triplicate.

### Isocitrate dehydrogenase (Rv3339c) *in vitro* assay

Isocitrate dehydrogenase (ICDH) activity was determined spectrophotometrically by monitoring the time dependent reduction of NADP^+^ to NADPH. Assays were performed at room temperature in a final reaction volume of 100 μL as described^12^. To determine modulation of ICDH1 activity by Rv2170, ICDH1 was pre-incubated with or without Rv2170 in a 100 μL volume of HEPES buffer (50 mM, pH 7.5) containing 200 μM acetyl-CoA for 2 hours at 30 °C. 5 μL of the reaction mixture was used for the isocitrate dehydrogenase assays. The ICDH assay mixtures contained 20 mM Tris-HCl buffer pH 7.5, 2 mM NADP^+^, 0.25 mM DL-isocitrate, 10 mM MgCl_2_, 5 mM MnCl_2_ and 2 μg of ICDH1. The assays were initiated by adding the preincubated ICDH1 and monitored using Perkin-Elmer EnVision microtiter plate reader at 340 nm. Triplicates for each set were performed.

### Mass spectrometry analysis

After *in vitro* acetylation, reaction mixture was resolved by SDS-PAGE. The ICDH1 band was excised from the gel and destained with wash solution (60% acetonitrile in 0.2 M ammonium bicarbonate, 0.1% TFA (trifluoroacetic acid), 1:1, v/v), and dehydrated. Dried gel pieces then were treated with 1 μg of a modified trypsin (Roche Molecular Biochemicals) overnight at 37 °C. The peptide mixtures were extracted from the gel with an extraction solution (60% acetonitrile in water, 0.1% TFA). The extract was pooled, dried and dissolved in buffer A (water : acetonitrile : acetic acid, 95:5:0.1 v/v). The sample was analyzed by nanoLC-MS/MS using *LTQ Orbitrap Velos Hybrid FT* (Thermo Scientific) at Cornell Proteomics and Mass Spectrometry Facility. The peptide mass data were processed with MASCOT (Matrix Science) against SwissProt Mtb complex database by using lysine acetylation as a variable modification. The identified putative peptide modifications were verified manually.

## Results and Discussion

### Identification of a metabolic regulator of carbon flux in Mtb

**Figure 1:**
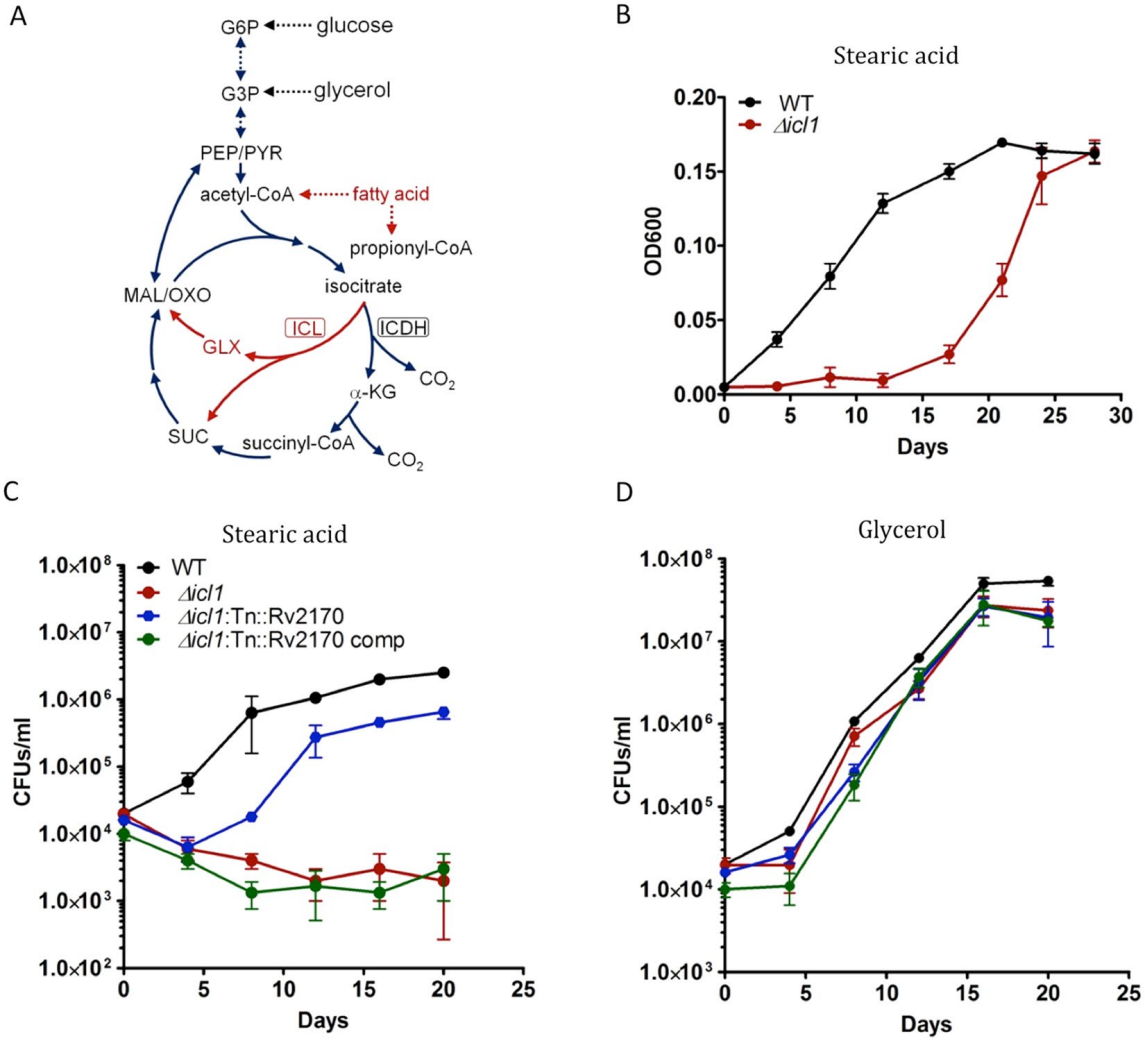
Mutant *Δicl1 Tn::Rv2170* restored growth on stearic acid. (**A**) Carbon flux from isocitrate is channeled either into the TCA cycle through isocitrate dehydrogenase (ICDH) activity or into the glyoxylate shunt through isocitrate lyase (ICL) activity. G6P, glucose-6-phosphate; G3P, glycerol-3-phosphate; PEP, phosphoenolpyruvate; PYR, pyruvate; MAL, malate; SUC, succinate; GLX, glyoxylate; α-KG, α-ketoglutarate. Activated metabolic pathways in presence of fatty acid as carbon source was indicated in red. (**B**) Bacterial growth was determined for wild-type H37Rv and the *Δicl1* strain in minimal medium containing 0.05 mM stearic acid as a sole carbon source. (**C** and **D**) Bacterial CFU (colony forming unit) counts were determined every 4 days for a 20 day period for wild type, *Δicl1* strain, *Δicl1:Tn::Rv2170* and complementation strain in minimal medium containing 0.05 mM stearic acid (**C**) or 10 mM glycerol (**D**). Error bars is standard deviation from triplicates.

The glyoxylate shunt is an anabolic pathway thought to be essential for the generation of TCA intermediates by converting acetyl-CoA to succinate to support growth on non-carbohydrate substrates (Fig. 1A)^22^. In Mtb, isocitrate lyase (ICL1, Rv0467), the first enzyme in the glyoxylate shunt is required for growth on fatty acid as sole carbon source^23^. Unexpectedly, as shown in Fig. 1B, we found that although the H37Rv *Δicl1* mutant was capable of growing in minimal medium containing stearic acid (a saturated C18 fatty acid) as a sole carbon source, active replication was subject to a long initial lag phase (~20 days). Such a phenotype is frequently generated by negatively regulating alternative metabolic pathways and these suppression circuits may be subject to inactivation by transposon mutagenesis thus restoring the wild-type growth phenotype.

To test this possibility, we constructed a transposon insertion library in a H37Rv *Δicl1* mutant background (1 × 10^5^ individual clones)^7^. We initially cultured this transposon library on liquid minimal medium containing 0.05 mM stearic acid as the sole carbon source for 7 days and then we expanded the culture in rich medium (Middlebrook 7H9 OADC), followed by plating on 7H10 OADC agar. We picked 32 transposon mutants that restored growth to the *Δicl1* mutant during the initial lag phase, and identified the transposon insertion sites by DNA sequencing (detailed in Supplementary Table 1). The most frequent insertions (27/32) among selected mutants inactivated Rv2170 which is annotated as a GNAT. We next compared the relative growth rates of the *Δicl1:Tn::Rv2170* mutant with the parent *Δicl1* mutant and WT Mtb in minimal medium containing 0.05 mM stearic acid as the sole carbon source. As shown in Fig. 1C, the *Δicl1:Tn::Rv2170* mutant grew faster than the *Δicl1* mutant without the long initial lag phase. We were able to restore this delayed growth phenotype by expressing a wild type allele of Rv2170 from a replicating plasmid, confirming that the growth delay phenotype was dependent on Rv2170. In addition, we tested the growth of all strains in non fatty acid medium containing 10 mM glycerol (Fig. 1D). As expected, the *Δicl1:Tn::Rv2170* mutant grew normally on glycerol, indicating that the *Δicl1:Tn::Rv2170* mutant does not have a general growth defect. These results are consistent with our original hypothesis that the growth delay was a suppressor phenotype and this suggested that Rv2170 plays a role in the regulation of carbon flow from the breakdown of stearic acid.

### Mutant *Δicl1 Tn::Rv2170* actively metabolizes stearic acid

**Figure 2:**
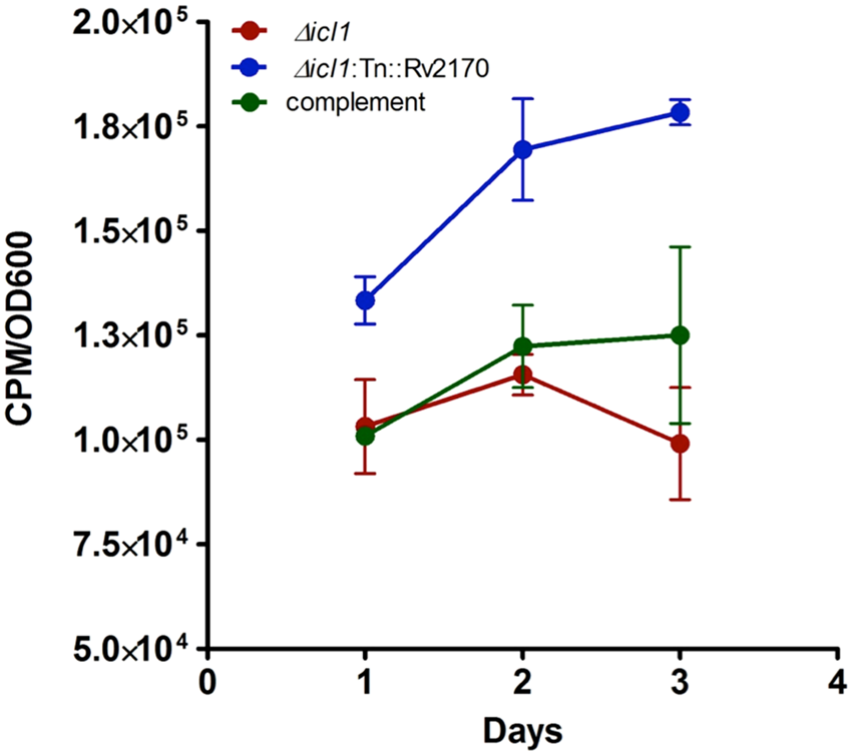
Inactivation of Rv2170 in the *Δicl1* mutant increases generation of CO_2_ from stearic acid. Metabolic activity of the bacterial cell was determined using conversion of stearic acid to CO_2_. Mtb strains were grown in minimal medium containing [1-^14^C] stearic acid and cold 0.05 mM stearic acid: *Δicl1* strain, *Δicl1:Tn::Rv2170* and complementation strain. CO_2_ released from culture was trapped with NaOH and the labeled CO_2_ was analyzed with a scintillation counter. The CPM data are shown at different time points. Data are representative from biological triplicates.

We next evaluated whether inactivation of Rv2170 in the *Δicl1* mutant background (*Δicl1:Tn::Rv2170*) leads to increased carbon utilization on stearic acid medium. We quantified stearic acid catabolism by measuring ^14^CO_2_ evolution from [1-^14^C]stearic acid^24^. The conversion of [1-^14^C]stearic acid to ^14^CO_2_ was assessed in minimal medium containing 0.05 mM stearic acid augmented with 1 μCi of [1-^14^C]stearic acid, and release of ^14^CO_2_ was measured over 3 days (Fig. 2). Inactivation of Rv2170 in the *Δicl1* mutant resulted in an increase in the rate of CO_2_ production which is consistent with the increased growth of the double mutant shown in Fig. 1C. Moreover, the enhanced metabolic activity in the Tn::Rv2170 mutant was reversed by expressing a wild type allele of the Rv2170 gene from a replicating plasmid. We conclude that Rv2170 suppresses Mtb’s ability to utilize stearic acid-derived carbons through the CO_2_ releasing stages of the TCA cycle and slows bacterial growth under this condition.

### *Rv2170* homologues are present throughout the *Corynebacterineae*

**Figure 3:**
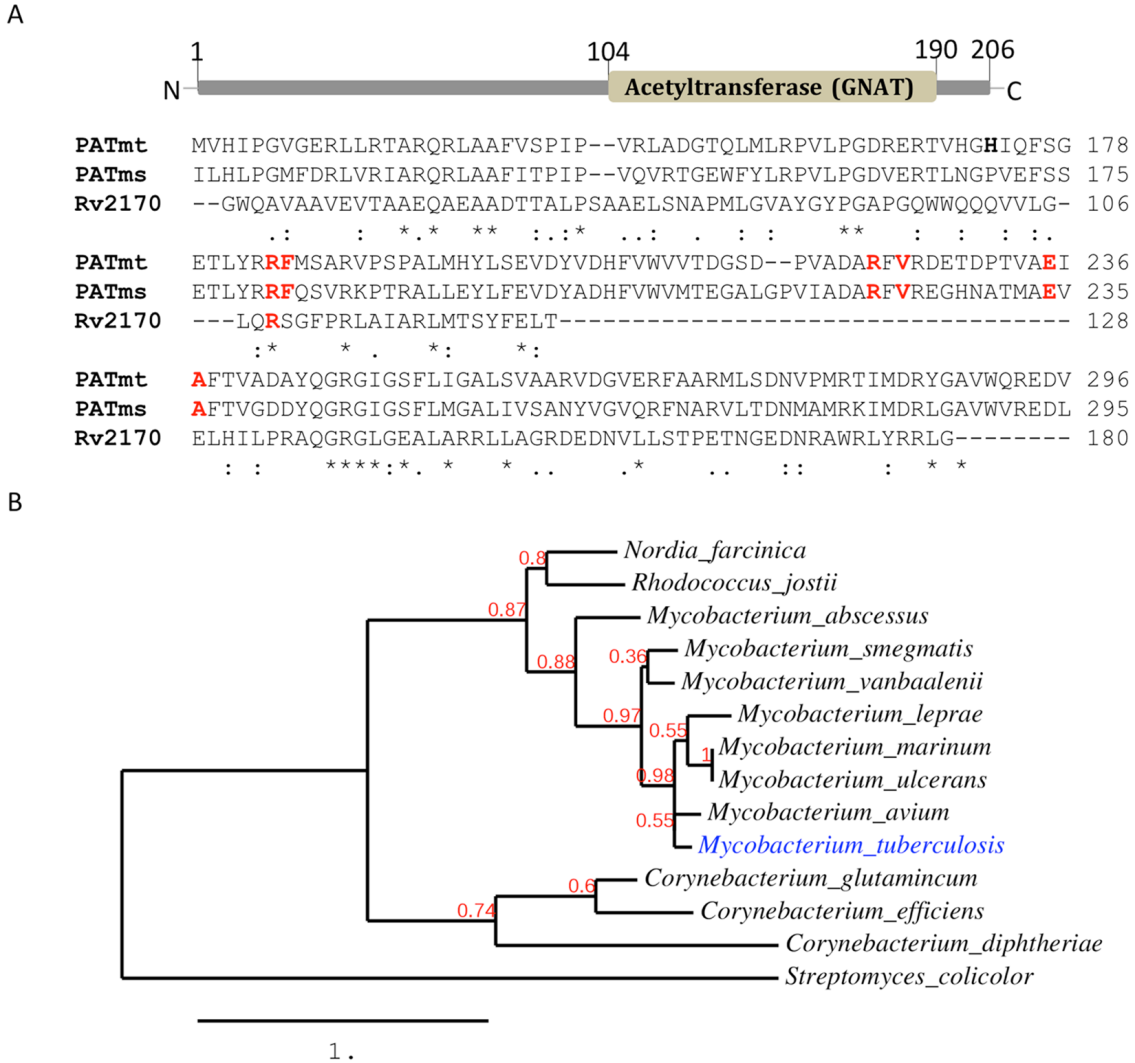
Rv2170 encodes GNAT acetyltransferase. (**A**) Multiple sequence alignment of the GNAT domain from mycobacterial acetyltransferases: Rv2170, PATmt (Rv0998:*M. tuberculosis* H37Rv), and PATms(MSMEG_5458: *M. smegmatis* MC2). Sequence alignment was performed using ClustalW2. Residues in the catalytic site are shown in red^44^. (**B**) Neighbor joining phylogenetic tree of Mtb Rv2170. Selected protein sequences from a blast search using the protein sequence of Rv2170 in the TB database (http://www.tbdb.org) were used as input for the phylogenetic tree. The tree was generated by using MUSCLE for alignment and PhyML for tree construction in Robust Phylogenetic Analysis interface^18^. The numerical values represent the branch support value.

*Rv2170* is predicted to encode a protein containing a GNAT domain in its C-terminal region. These enzymes commonly catalyze the acetylation of the epsilon amino group of lysine residues in target proteins^25^. It is known that *N*-Lysine acetylation, a ubiquitous post-translational modification, can regulate central cellular processes such as carbon metabolism and transcriptional regulation^26–28^. The GNAT domain coded by *Rv2170* (22.9 kDa, predicted size, 206 amino acids) shares only 32% amino acids with the GNAT domain of a known Mtb protein acetyltransferase Rv0998^29^. In addition, Rv2170 lacks the cofactor binding domain for cAMP present in Rv0998 (Fig. 3A)^29^. Further comparison reveals that only Arg184 among 6 residues in catalytic site for transferring acetyl group in Rv0998 is conserved in Rv2170, raising possibility that Rv2170 may catalyze acetylation through a different mechanism. Blast analysis against the NCBI protein database using the amino acid sequence of the Rv2170 revealed that the Rv2170 is highly conserved in all *Mycobacterium* species, and clusters with other gram positive bacteria including *Corynebacterium* (*C. efficines, C. diphtheria, and C. jeikeium;* 37–45% identities), *Rhodococcus* (*R. jostii RHA1;* 53% identity)*,* and *Nocarida* (*N. farcinia;* 53% identity) (Fig. 3B).

### The acetyltransferase encoded by Rv2170, acetylates isocitrate dehydrogenase 1 (Rv3339c)

Rv2170 mediates the reduced production of CO_2_ from stearic acid in a *Δicl1* background. There are two potential mechanisms underpinning this phenotype. Firstly, the *Δicl1* mutant could open the methylmalonyl pathway as a alternative pathway for acetyl-CoA^30^. However, we think this unlikely since the methylmalonyl-CoA mutase is dependent on exogenous vitamin B12 as a cofactor^31^. An alternative, more favored explanation is that Rv2170 regulates a key node of central carbon metabolism, such as isocitrate dehydrogenase (ICDH) activity. ICDH acts at the branching node between glyoxylate shunt and TCA cycle. ICDH catalyzes conversion of isocitrate and NADP^+^ into the TCA intermediate α-ketoglutarate and NADPH. Thus, if Rv2170 was involved in the down-regulation of ICDH activity, the direction of carbon flow could be switched toward the TCA cycle in the *Δicl1:Tn::Rv2170* mutant, which would lead to enhanced generation of CO_2_ through the conversion of α-ketoglutarate into succinate and CO_2_ by α-ketoglutarate dehydrogenase activity. In addition to the generation of α-ketoglutarate, it is also important to note that during growth on short chain fatty acid such as acetate, ICDH generates 90% of the NADPH required for biosynthetic pathways^32^.

*E. coli* and gram-negative bacteria, regulate ICDH activity through phosphorylation by AceK^33, 34^. In contrast, in eukaryotes, mitochondrial ICDH activity is regulated by acetylation of ICDH by lysine acetyltransferases^28, 35^. Mtb encodes two structurally distinct NADP^+^-dependent ICDHs; Rv3339c (ICDH1) and Rv0066c (ICDH2)^12^. ICDH2 (82.5 kDa) larger than ICDH1 (45.5 kDa) and is more similar to prokaryotic ICDH in having two catalytic sites. Interestingly, the Mtb ICDH1 is structurally closer to the mitochondrial ICDH; and the mitochondrial ICDH has six lysine residues that can be acetylated and these lysine residues are highly positionally-conserved in Mtb ICDH1. This suggests that the Mtb ICDH1 may be regulated by acetylation rather than phosphorylation to modulate its activity.

**Figure 4:**
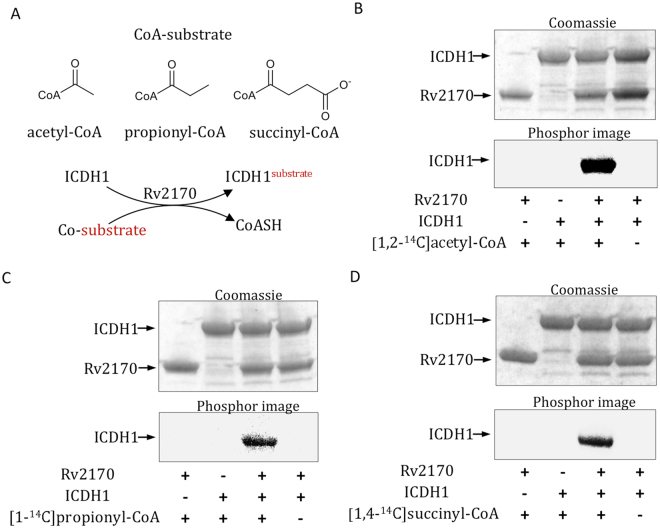
Rv2170 modifies ICDH1 using CoA substrates from TCA cycle and methylcitrate cycle. (**A**) The protein acetyltransferase Rv2170 modifies ICDH1 by using CoA substrates, acetyl-CoA, succinyl-CoA, and propionyl-CoA. (**B**–**D**) ICDH1 was incubated with [1,2-^14^C] acetyl-CoA (**B**), [1-^14^C] propionyl-CoA (**C**), or [1,4-^14^C]succinyl-CoA (**D**) in the presence or absence of Rv2170. Proteins were resolved by SDS-PAGE and visualized with Coomassie Blue. Acetylation of proteins was detected by phosphor image.

To test whether Rv2170 modifies Mtb ICDH1 and/or ICDH2 through acetylation, we cloned and overproduced all three proteins as C-terminal His-tag fusion proteins in *E. coli*. *In vitro* acetyltransferase assays were performed with Rv2170, ICDH1 & 2 and the [1, 2-^14^C] acetyl-CoA donor (Fig. 4A). As shown in Fig. 4B, we found that Mtb ICDH1 was robustly acetylated by Rv2170; in contrast, ICDH2 remained unlabeled (data not shown). In addition, to determine whether Rv2170 is able to modify ICDH1 with other donors, we tested succinyl-CoA and propionyl-CoA, which are provided via the TCA cycle and methylcitrate cycle, respectively. Interestingly, as shown in Fig. 4C and D, we found that Rv2170 is also capable of modifying ICDH1 with the succinyl-CoA and propionyl-CoA donors. This is consistent with the expanded substrate specificity exhibited by GNAT-like acyltransferases^36^. These data suggest that Rv2170 might play a role in regulating the TCA cycle and methylcitrate cycle when Mtb utilizes fatty acid as carbon source. Furthermore, to test whether possible cofactors may modulate ICDH1 acetylation, we assessed the impact of adding cAMP and NADH to the acetylation assay. Unlike Rv0998c^29^, the addition of either cAMP or NADH to the reaction did not substantially change the ICDH1 acetylation reaction, which is consistent with lack of any recognizable cAMP regulatory domain in Rv2170 (Supplementary Figure 1).

### Rv2170 acetylates isocitrate dehydrogenase-1 at Lys30 and Lys129

**Figure 5:**
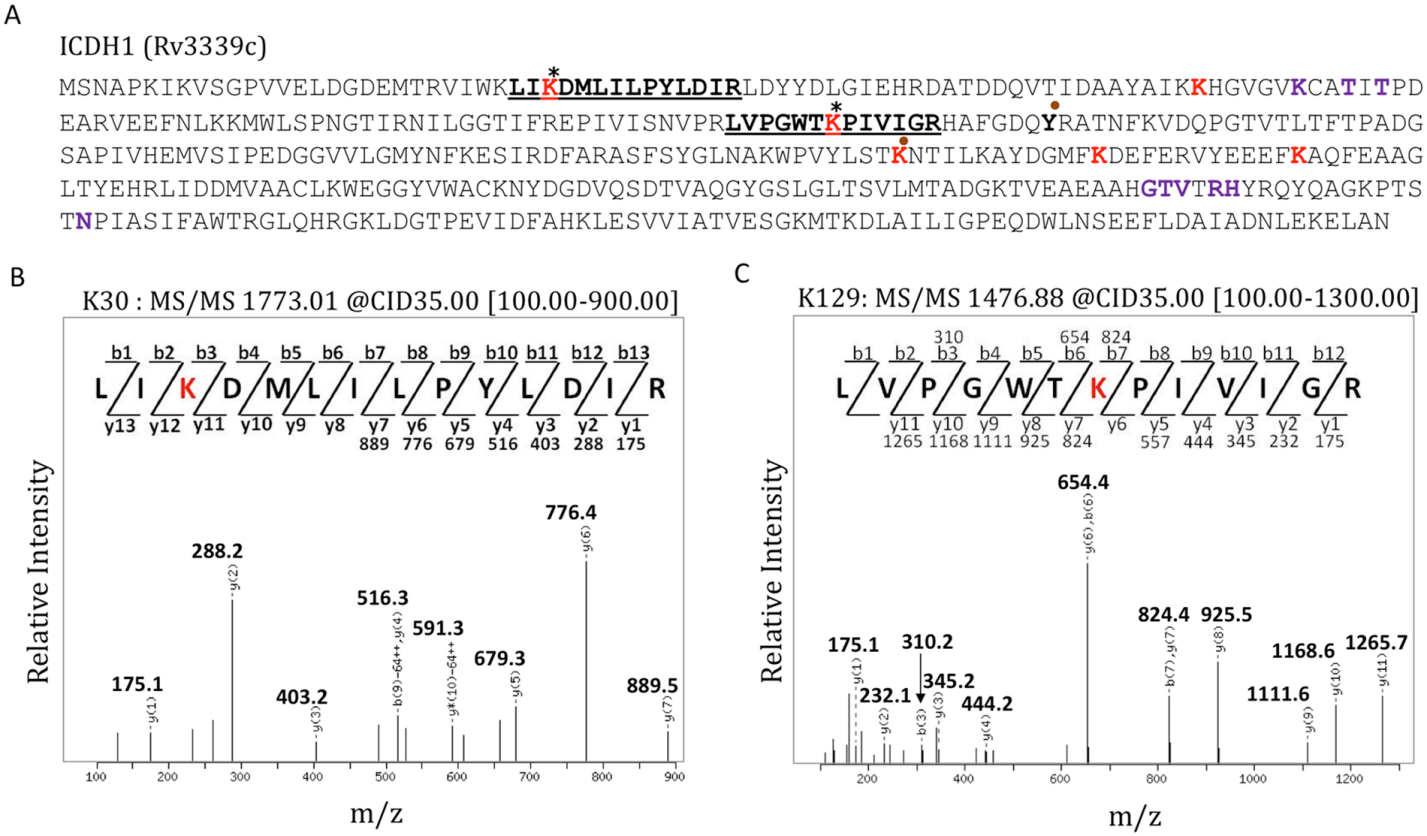
Rv2170 acetylates Mtb ICDH1 (Rv3339c) at K30 and K129. (**A**) Protein sequence of ICDH1. six lysine residues in both Mtb ICDH1 and human mitochondrial ICDH known to be acetylated are shown in red are conserved^45^. The two acetylated lysine residues in Mtb ICDH1 detected in our study are marked with asterisk. Substrate binding regions for isocitrate and NADP^+^ are highlighted with dot and purple respectively. (**B** and **C**) modified sites of ICDH1 by Rv2170 were identified by mass spectrometry analysis. The MS/MS spectrum of acetylated peptides LI**K**DMLILPYLDIR and LVPGWT**K**PIVIGR of ICDH1 were shown and the observed fragment ions (b and y ions) are marked on the spectrum and also summarized schematically. These peptides are underlined on the protein sequence (**A**).

To determine the location and number of acetyl groups transferred to ICDH1 by Rv2170, ICDH1 was acetylated *in vitro*, purified, digested with trypsin, and analyzed by LC-MS/MS. The mass spectrometry analysis revealed that ICDH1 was acetylated at residues Lys30 and Lys129 (Fig. 5). The two signals, at m/z = 1773.01 and at m/z = 1476.88 corresponded to the LI**K**DMLILPYLDIR and LVPGWT**K**PIVIGR peptides of ICDH1 modified with a single acetyl group, respectively (Supplementary Figure 2). These sites of acetylation lie outside the catalytic pocket of ICDH1; Lys215, which lies within the isocitrate binding pocket, was not modified by Rv2170. Active ICDH1 is a dimer^12, 37^ and we hypothesize that acetylation may impact activity through modulating higher order structure of the enzyme complex. These data, coupled with the absence of an AceK homologue in Mtb, suggests that the regulation of Mtb ICDH1 is unlikely to undergo in similar mechanism to *E. coli* ICDH. Furthermore, in ICDH of *E. coli* AceK phosphorylates the protein at Ser113, which is the binding site for isocitrate, and the Mtb ICDH1 lacks a comparable acceptor residue^37–39^.

### Activity of isocitrate dehydrogenase Rv3339c is repressed by acetylation by Rv2170

**Figure 6:**
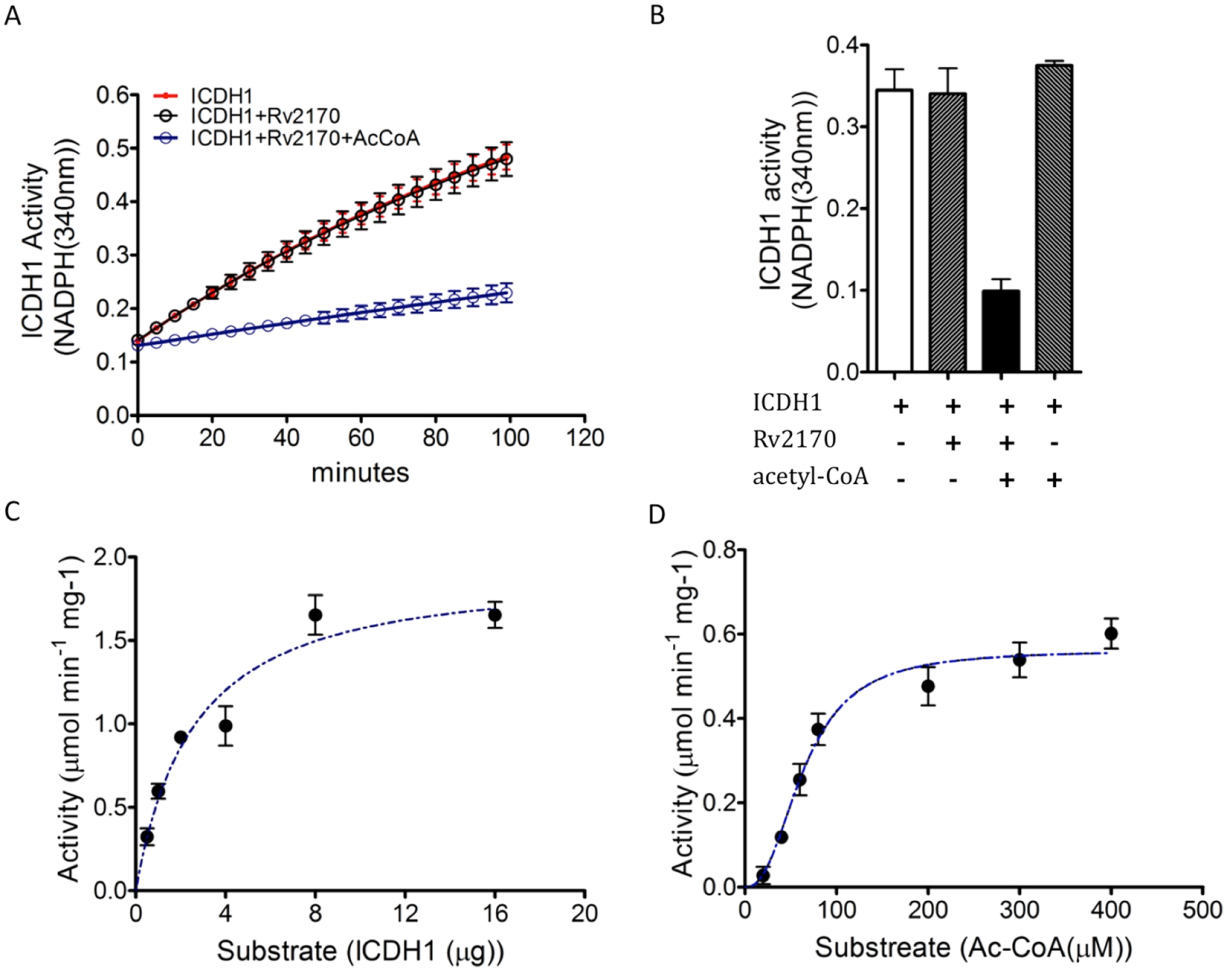
ICDH1 activity is repressed through acetylation by Rv2170. (**A** and **B**) ICHD1 activity was measured by quantifying NADPH generation during incubation in the presence or absence of Rv2170 and acetyl-CoA. ICDH1 activity was inhibited by Rv2170 only in the presence of acetyl-CoA. (**C** and **D**) Graphs show substrate saturation curves for lysine acetylation by Rv2170 (**C**) as the ICDH1 substrate is varied at a saturating concentration of acetyl-CoA(200 μM) or (**D**) as the acetyl-CoA substrate is varied at a saturating concentration of ICDH1 (10 μg) Error bars show standard deviations from three representative triplicate.

To test the functional consequences of the acetylation of ICDH1 by Rv2170 we performed an *in vitro* ICDH assay to examine ICDH1 activity following pre-incubation with Rv2170. The ICDH activity was monitored by NADPH production at 340 nm. As shown in Fig. 6A and B, while the acetylation by Rv2170 did not abolish the activity of ICDH1 completely, we found that activity decreased to around 30% that of unmodified ICDH1 following incubation with Rv2170. Comparable partial loss of activity has been observed in many acetylated enzymes^40^. It should be noted that *E. coli* ICDH retains considerable activity even after post translational modification by AceK and during growth on acetate, and ~25% of ICDH is still un-phosphorylated and active^41^. Taken together, the genetic screen and biochemical data support the conclusion that *Rv2170* of Mtb encodes an actyltransferase enzyme capable of modulating the activity of the central metabolic enzyme ICDH1 through lysine acetylation.

### Assessment of enzymatic activity and substrate cooperativity for Rv2170 acetyltransferase

To characterize the acetylation reaction catalyzed by Rv2170 further, we developed a coupled enzymatic assay to monitor the acetylation reaction continuously. The acetyltransferase activity of Rv2170 was assayed by quantifying the formation of 2-nitro-5-thiobenzoate (TNB2) at 412 nm^21^. The enzyme assay showed a typical hyperbolic response for various ICDH1 concentrations at a saturating concentration of 200 μM acetyl-coA (Fig. 6C). However, we observed a sigmoidal response in kinetic analysis of Rv2170 when the concentration of acetyl-CoA was increased at a fixed ICDH1 acceptor concentration of 10 μg (Fig. 6D). These data suggest that acetylation on ICDH1 by Rv2170 is unlikely to be cooperative at unsaturated concentration of ICDH1 (<10 μg) although Rv2170 responds differentially to acetyl-CoA at the saturated concentration of ICDH1.

### The Rv2170 deficient mutant lacks a growth phenotype in wild-type Mtb background

Since mutations in *rv2170* reverse the growth delay in the *Δicl1* double mutant strain we sought to characterize the phenotype of a *rv2170* single mutant. We constructed a clean Rv2170 deletion strain in H37Rv as described^42^, and evaluated growth of the *ΔRv2170* mutant on various carbon sources including glucose, glycerol, acetate, and stearic acid. Inactivation of Rv2170 led to no general growth defects, and the *ΔRv2170* mutant grew to levels comparable to WT on all carbon sources including stearic acid (Supplementary Figure 3). Thus, inactivation of Rv2170 is only beneficial for bacterial growth in the absence of ICL. We speculate that growth of the *ΔRv2170* single mutant on stearic acid and acetate occurs because the *ΔRv2170* mutant has a functional ICL enzyme. Activation of ICL produces glyoxylate, which may potentially cross-activate the ICDH2 enzyme as recently reported in mycobacteria^43^. Stimulating ICDH2 activity with glyoxylate could conceivably maintain the balance of carbon flow through the oxidative TCA cycle and the anaplerotic reactions allowing bacterial growth. Lastly, given that inactivating Rv2170 confers a growth benefit by reversing a lag in growth in the *Δicl1* background, it is not surprising that the single *ΔRv2170* mutant growth phenotype is minimal. These data further indicate that the ICL enzyme confers considerable metabolic flexibility for Mtb and provides additional appreciation of the multiple roles this enzyme plays in balancing carbon metabolism.

## Conclusions

**Figure 7:**
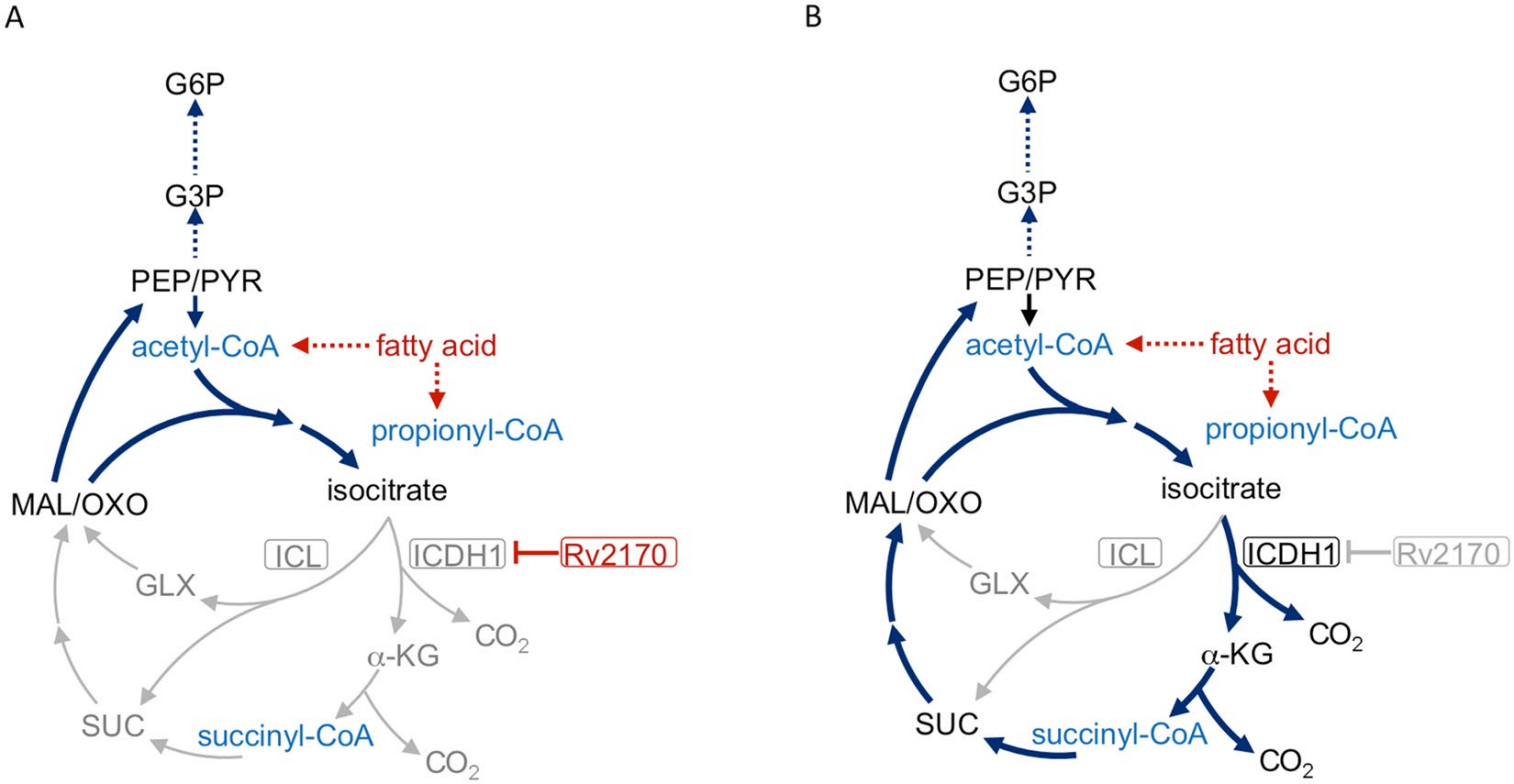
Carbon metabolism modulation by Rv2170. A diagram modeling the “preferred” metabolic fate of carbon at the metabolic node isocitrate. Rv2170, an ICDH1 acetyltransferase modulates carbon flow to either closing (**A**) or opening (**B**) toward TCA cycle during growth on stearic acid as sole carbon substrate. The direction of the dominant carbon flow is indicated by black instead of grey arrows. The CoA substrates utilized by Rv2170 are shown in blue. Symbols are as in Fig. 1A.

The regulation of carbon metabolism is an essential part of the metabolic flexibility of Mtb which enables the bacterium to exploit host fatty acid and lipids as primary carbon sources during infection. In the current study, we took advantage of an Mtb *Δicl1* mutant, which lacks both the glyoxylate shunt and the methylcitrate cycle to channel carbon flux downstream of isocitrate. The mutant strain exhibited a delayed growth phenotype in stearic acid that allowed us to perform a genetic screen to identify suppressors of stearic acid utilization. The screen identified a novel protein acetyltransferase encoded by *Rv2170*. Further analysis of Rv2170 demonstrated that the enzyme acetylated ICDH1 leading to a reduction in its activity. Our data demonstrate that, unlike most bacteria, Mtb employs acetylation instead of phosphorylation for regulation of ICDH1 activity. In the presence of fatty acids, the repression of ICDH1 activity results in reduced carbon flow into the TCA cycle and reduced production of CO2 from stearic acid (Fig. 7A), while de-repression of ICDH1 experimentally through the loss of Rv2170 restores carbon flow from fatty acids into the TCA cycle (Fig. 7B). These data provide further insights into the regulatory pathways that underpin *Mycobacterium tuberculosis*’s metabolic flexibility at critical nodes of carbon flux.

## Electronic supplementary material

Supplementary Information
