# Supplementary material for: Novel protein acetyltransferase, Rv2170, modulates carbon and energy metabolism in Mycobacterium tuberculosis
Source: Sci Rep. 2017 Mar 6;7:72. doi: 10.1038/s41598-017-00067-1 (PMC5428333; doi:10.1038/s41598-017-00067-1)
Supplement: Supplementary file 1 — Supplementary Information [file 41598_2017_67_MOESM1_ESM.pdf]

Supplementary Information

Novel protein acetyltransferase, Rv2170, modulates carbon and energy metabolism in *Mycobacterium tuberculosis*

Wonsik Lee[1,2], Brian VanderVen[1], Suzanne Walker[2] and David G. Russell[1]*

[1]Department of Microbiology and Immunology, College of Veterinary Medicine, Cornell University, Ithaca, NY 14850, United States

[2]Department of Microbiology and Immunology, Harvard Medical School, Boston, Massachusetts, 02115, United States

*Correspondence:

David G. Russell

dgr8@cornell.edu

tel 607 253 4272

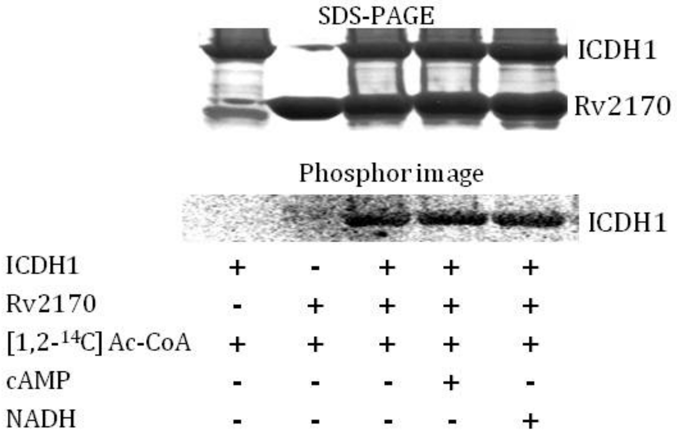

Supplementary Figure 1. Rv2170 acetylates ICDH1 independent on cAMP or NADH
ICDH1 was incubated with [1, 2-^{14}C] acetyl-CoA and Rv2170 in the presence of 1mM cAMP or 1mM NADH. Proteins were resolved in SDS-PAGE and visualized with Coomassie Blue. Acetylation of proteins was detected by phosphor image.

A fragment matches of peptide LIK(acetyl)DMLILPYLDIR

#	b	b^{++}	b*	b^{*++}	b^0	b^{0++}	Seq.	y	y^{++}	y*	y^{*++}	y^0	y^{0++}	#
1	114.0913	57.5493					L							14
2	227.1754	114.0913					I	1596.9360	798.9716	1579.9094	790.4583	1578.9254	789.9663	13
3	397.2809	199.1441	380.2544	190.6308			K	1483.8519	742.4296	1466.8253	733.9163	1465.8413	733.4243	12
4	512.3079	256.6576	495.2813	248.1443	494.2973	247.6523	D	1313.7464	657.3768	1296.7198	648.8635	1295.7358	648.3715	11
5	595.3450	298.1761	578.3184	289.6629	577.3344	289.1709	M	1198.7194	599.8633	1181.6929	591.3501	1180.7089	590.8581	10
6	708.4291	354.7182	691.4025	346.2049	690.4185	345.7129	L	1115.6823	558.3448	1098.6558	549.8315	1097.6717	549.3395	9
7	821.5131	411.2602	804.4866	402.7469	803.5026	402.2549	I	1002.5982	501.8028	985.5717	493.2895	984.5877	492.7975	8
8	934.5972	467.8022	917.5706	459.2890	916.5866	458.7969	L	*889.5142*	445.2607	872.4876	436.7475	871.5036	436.2554	7
9	1031.6499	**516.3286**	1014.6234	507.8153	1013.6394	507.3233	P	*776.4301*	388.7187	759.4036	380.2054	758.4196	379.7134	6
10	1194.7133	597.8603	1177.6867	589.3470	1176.7027	588.8550	Y	*679.3774*	340.1923	662.3508	331.6790	661.3668	331.1870	5
11	1307.7973	654.4023	1290.7708	645.8890	1289.7868	645.3970	L	*516.3140*	258.6606	499.2875	250.1474	498.3035	249.6554	4
12	1422.8243	711.9158	1405.7977	703.4025	1404.8137	702.9105	D	*403.2300*	202.1186	386.2034	193.6053	385.2194	193.1133	3
13	1535.9083	768.4578	1518.8818	759.9445	1517.8978	759.4525	I	*288.2030*	144.6051	271.1765	136.0919			2
14							R	*175.1190*	88.0631	158.0924	79.5498			1

B. fragment matches of peptide LVPGWTK(acetyl)PIVIGR

#	b	b^{++}	b*	b^{*++}	b^0	b^{0++}	Seq.	y	y^{++}	y*	y^{*++}	y^0	y^{0++}	#
1	114.0913	57.5493					L							13
2	213.1598	107.0835					V	1364.8049	682.9061	1347.7783	674.3928	1346.7943	673.9008	12
3	**310.2125**	155.6099					P	*1265.7365*	633.3719	1248.7099	624.8586	1247.7259	624.3666	11
4	367.2340	184.1206					G	*1168.6837*	584.8455	1151.6572	576.3322	1150.6731	575.8402	10
5	553.3133	277.1603					W	*1111.6622*	556.3348	1094.6357	547.8215	1093.6517	547.3295	9
6	**654.3610**	327.6841			636.3504	318.6788	T	*925.5829*	463.2951	908.5564	454.7818	907.5724	454.2898	8
7	**824.4665**	412.7369	807.4400	404.2236	806.4559	403.7316	K	*824.5353*	412.7713	807.5087	404.2580			7
8	921.5193	461.2633	904.4927	452.7500	903.5087	452.2580	P	*654.4297*	327.7185	637.4032	319.2052			6
9	1034.6033	517.8053	1017.5768	509.2920	1016.5928	508.8000	I	557.3770	279.1921	540.3504	270.6788			5
10	1133.6717	567.3395	1116.6452	558.8262	1115.6612	558.3342	V	*444.2929*	222.6501	427.2663	214.1368			4
11	1246.7558	623.8815	1229.7293	615.3683	1228.7452	614.8763	I	*345.2245*	173.1159	328.1979	164.6026			3
12	1303.7773	652.3923	1286.7507	643.8790	1285.7667	643.3870	G	*232.1404*	116.5738	215.1139	108.0606			2
13							R	*175.1190*	88.0631	158.0924	79.5498			1

Supplementary Figure 2. Mass spectrometry analysis of the acetylated peptide of Rv3339c
(A) fragment matches of peptide LIK(acetyl)DMLILPYLDIR. (B) Fragment matches of peptide LVPGWTK(acetyl)PIVIGR. Prominent picks in Figure 5B and 5C are shown in red.

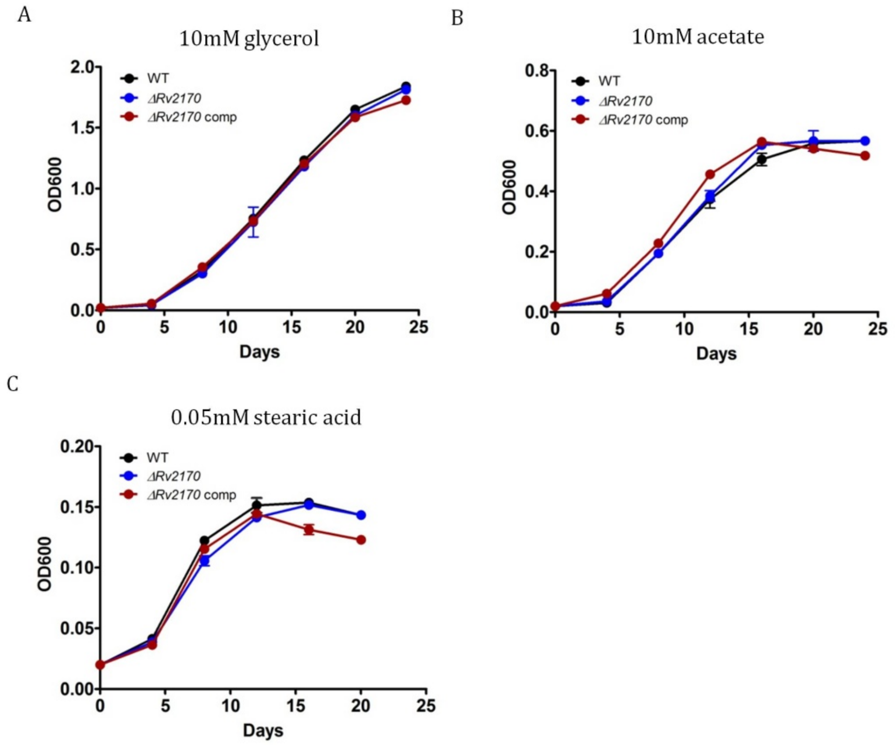

Supplementary Figure 3. Growth of the Rv2170 mutant in various carbon sources.
Bacterial growth was measured for the H37Rv wild type, *ΔRv2170*, and complementary strain in minimal medium containing 10 mM glycerol, 10 mM acetate (B), or 0.05 mM stearic acid (C) as a sole carbon source. Standard deviations were calculated from triplicate.

Supplementary Table 1. transposon mutants selected from the screen

gene	function	insertion Site (size of orf)	number of mutants
Rv2170	GCN5-related N-acetyltransferase	-199(621bp)	27
Rv3312A	Unknown	64 (312bp)	2
Rv0917	High-affinity uptake of glycine betaine	1291(1782bp)	1
Rv0645	Methoxy mycolic acid synthase 1	94(862bp)	1
Rv3757c	ABC transporter ProW	176(861bp)	1

Supplementary Table 2. Bacterial strains and plasmids used in this study

strains or plasmids	characteristics [a]	ref or source
Plasmid		
pET23a	6xHis–tag fusion protein expression vector; amp^R	Novagen
pET23a-Rv2170	pET-23a-Rv2170; amp^R	This study
pET23a-Rv3339c	pET-23a-Rv2170; amp^R	This study
pVV16	protein expression vector for Mtb; km^R, hyg^R	42
pVV16-Rv2170	pVV16-Rv2170; ampicillin resistant , hyg^R	This study
pYUB854	gene knockout vector; *rpsL*(wt), hyg^R	42
pYUB854-*ΔRv2170*	pYUB854- **Δ**Rv2170; hyg^R	This study
E.coli		
BL21(DE3)	*F- ompT hsdS* ($r_B^- m_B^-$) *gal dcm*	Novagen
TOP 10	*F- mcrA Δ(mrr-hsdRMS-mcrBC) φ80lacZΔM15 ΔlacX74 nupG recA1 araD139 Δ(ara-leu)7697 galE15 galK16 rpsL(Str^R) endA1 λ^-*	Invitrogen
M. tuberculosis		
H37Rv wt		Lab collection
H37Rv *rpsL*	rpsL(mut), $strep^R$	Lab collection
H37Rv *Δicl1*	***Δicl1*** (Rv0467), hyg^R	17
H37Rv *Δicl1* :: Tn:2170	***Δicl1*** :: Tn 2170, km^R, hyg^R	This study
H37Rv *ΔRv2170*	hyg^R	This study
H37Rv *ΔRv2170* / pVV16-Rv2170	km^R, hyg^R	This study

[a]: ampR: ampicillin resistant; km^R : kanamycin resistant; hyg^R: hygromycin resistant; $strep^R$: streptomycin resistant

Supplementary Table 3. Primers used in this study

name	oligonucleotide Sequence (5'→3')[b]	use
Rv2170 CA	CGAGCGTAGGCGTCGGTGACAAA	Deletion verification
Rv2170 CB	CCGAAGCTGGATGTACTCGATTTCTGGT	Deletion verification
Rv2170 AA	CC**AAGCTT**CCAAGAACTGGAGTCTCCGGACATGC	Rv2170 deletion(pYUB854)
Rv2170 AB	AAC**GCTAGC**CAACGCGCGGTGTCCTCCTCA	Rv2170 deletion(pYUB854)
Rv2170 BA	TAC**TCTAGA** GCACGCTACCGCTCTAACCCGC	Rv2170 deletion(pYUB854)
Rv2170 BB	CTC**GGTACC** GAGACCTTCGCGGAAATGCTTGC	Rv2170 deletion(pYUB854)
Rv2170-orf-F	TAA**CATATG**TTAGAGCGGTAGCGTGCGACCCA	Rv2170 expression (pET23a, pVV16)
Rv2170-orf-R	CC**AAGCTT** TTAGAGCGGTAGCGTGCGACCCA	Rv2170 complementation (pVV16)
Rv2170-orf-Rs	CC**AAGCTT** GAGCGGTAGCGTGCGACCCA	Rv2170 expression(pET23a)
ICDH1-orf-F	TAA**CATATG**TCCAACGCACCCAAGATAAAA	ICDH1 expression(pET23a)
ICDH1-orf-R	CC**AAGCTT** ATTGGCCAGCTCCTTTTCCAGGTT	ICDH1 expression(pET23a)

[b]: underbars: restriction enzyme cleavage sites
